# Distribution of GC-rich heterochromatin and ribosomal genes in three fungus-farming ants (Myrmicinae, Attini, Attina): insights on chromosomal evolution

**DOI:** 10.3897/compcytogen.v15.i4.73769

**Published:** 2021-11-25

**Authors:** Gisele Amaro Teixeira, Luísa Antônia Campos Barros, Hilton Jeferson Alves Cardoso de Aguiar, Denilce Meneses Lopes

**Affiliations:** 1 Programa de Pós-graduação em Biologia Celular e Estrutural, Universidade Federal de Viçosa, Viçosa, MG 36570-900, Brazil; 2 Laboratório de Citogenética de Insetos, Departamento de Biologia Geral, Universidade Federal de Viçosa, Viçosa, MG 36570-900, Brazil; 3 Universidade Federal do Amapá, Campus Binacional, n°3051, Bairro Universidade, 68980-000, Oiapoque, Amapá, Brazil

**Keywords:** Biodiversity, chromatin, chromosomal rearrangements, Formicidae, karyotype evolution, molecular cytogenetics

## Abstract

Cytogenetic studies on fungus-farming ants have shown remarkable karyotype diversity, suggesting different chromosomal rearrangements involved in karyotype evolution in some genera. A notable cytogenetic characteristic in this ant group is the presence of GC-rich heterochromatin in the karyotypes of some ancient and derivative species. It was hypothesized that this GC-rich heterochromatin may have a common origin in fungus-farming ants, and the increase in species studied is important for understanding this question. In addition, many genera within the subtribe Attina have few or no cytogenetically studied species; therefore, the processes that shaped their chromosomal evolution remain obscure. Thus, in this study, we karyotyped, through classical and molecular cytogenetic techniques, the fungus-farming ants *Cyphomyrmextransversus* Emery, 1894, *Sericomyrmexmaravalhas* Ješovnik et Schultz, 2017, and *Mycetomoelleriusrelictus* (Borgmeier, 1934), to provide insights into the chromosomal evolution in these genera and to investigate the presence the GC-rich heterochromatin in these species. *Cyphomyrmextransversus* (2n = 18, 10m + 2sm + 6a) and *S.maravalhas* (2n = 48, 28m + 20sm) showed karyotypes distinct from other species from their genera. *Mycetomoelleriusrelictus* (2n = 20, 20m) presented the same karyotype as the colonies previously studied. Notably, *C.transversus* presented the lowest chromosomal number for the genus and a distinct karyotype from the other two previously observed for this species, showing the existence of a possible species complex and the need for its taxonomic revision. Chromosomal banding data revealed GC-rich heterochromatin in all three species, which increased the number of genera with this characteristic, supporting the hypothesis of a common origin of GC-rich heterochromatin in Attina. Although a single chromosomal pair carries rDNA genes in all studied species, the positions of these rDNA clusters varied. The rDNA genes were located in the intrachromosomal region in *C.transversus* and *M.relictus*, and in the terminal region of *S.maravalhas*. The combination of our molecular cytogenetic data and observations from previous studies corroborates that a single rDNA site located in the intrachromosomal region is a plesiomorphic condition in Attina. In addition, cytogenetic data obtained suggest centric fission events in *Sericomyrmex* Mayr, 1865, and the occurrence of inversions as the origin of the location of the ribosomal genes in *M.relictus* and *S.maravalhas*. This study provides new insights into the chromosomal evolution of fungus-farming ants.

## Introduction

Fungus-farming ants, included in the subtribe Attina (sensu Ward et al. 2015), have an obligatory symbiotic relationship with fungi (Weber 1966). In this symbiosis, these ants cultivate the fungus for food and, in return, provide the fungus with nutrition, propagate it to new locations, and protect it against parasitic microorganisms (Weber 1966; Little et al. 2005). In this agricultural system, these ants use different types of substrates depending on the genus/species (reviewed by Mehdiabadi and Schultz 2010), and with this, they play important roles in natural ecosystems, such as dispersion and increasing the success of seed germination, soil structuring, and nutrient cycling (Leal and Oliveira 1998; Fernandez-Bou et al. 2019).

Several molecular phylogenetic studies have been conducted in Attina to address the relationships between genera and species (Schultz and Brady 2008; Ješovnik et al. 2017; Sosa-Calvo et al. 2017; Solomon et al. 2019). These phylogenies support the monophyly of the group, with an origin of approximately 50–60 million years ago (Schultz and Brady 2008; Nygaard et al. 2016; Sosa-Calvo et al. 2017). This group includes approximately 280 described taxa distributed in 20 genera (Bolton 2021), which are grouped into two monophyletic sister clades: Paleoattina (*Apterostigma* Mayr, 1865, *Mycocepurus* Forel, 1893, and *Myrmicocrypta* Smith, 1860) and Neoattina (the remaining 17 genera) (Sosa-Calvo et al. 2018; Solomon et al. 2019; Cristiano et al. 2020).

Some Attina genera have been extensively revised (Sosa-Calvo et al. 2017, 2018; Solomon et al. 2019; Cristiano et al. 2020), and in this scenario, cytogenetics is a tool that can help in taxonomic issues, since chromosomal rearrangements can lead to reduced gene flow between populations and reproductive isolation, playing an important role in speciation (Riesemberg 2001; reviewed by Faria and Navarro 2010). In addition to evolutionary, phylogenetic, and chromosomal patterns in different groups, cytogenetic studies on ants, using classical and molecular techniques, are important for the understanding of taxonomically challenging species (Mariano et al. 2012; Santos et al. 2016; Aguiar et al. 2017; Micolino et al. 2019a; Teixeira et al. 2021).

Cytogenetic data are available for 56 taxa of fungus-farming ants with representatives from 12 genera (reviewed by Mariano et al. 2019; Aguiar et al. 2020; Micolino et al. 2020; Barros et al. 2021) and the chromosome number observed for the group ranges from 2n = 8 in *Mycocepurusgoeldii* (Forel, 1893) and *Mycocepurus* sp. to 2n = 64 in *Mycetophylaxlectus* (Forel, 1911) (as *Cyphomyrmexlectus*) (reviewed by Mariano et al. 2019). A notable cytogenetic characteristic in this ant group is that some Paleoattina and Neoattina species have GC-rich heterochromatin in all chromosomes, with nucleotide composition yet to be determined but may have an origin in the common ancestor needing further investigation (Barros et al. 2018; reviewed by Mariano et al. 2019). Molecular cytogenetic studies using fluorescence *in situ* hybridization (FISH) for mapping ribosomal genes have already been performed in 17 taxa, including six genera showing a single chromosome pair carrying rDNA genes (reviewed in Teixeira et al. 2021).

According to available cytogenetic data, different chromosomal rearrangements have been proposed to explain karyotype evolution in some Attina genera. The occurrence of centric fissions, according to Minimum Interaction Theory (MIT) (Imai et al. 1994), was suggested to explain the remarkable karyotype variation in *Mycetarotes* Emery, 1913 (2n = 14 to 54), *Apterostigma* (2n = 20 to 46), *Cyphomyrmex* Mayr, 1862 (2n = 20 to 42), and in leaf-cutting ants, in which *Amoimyrmexstriatus* (Roger, 1863) and *Atta* spp. present 2n = 22, and most *Acromyrmex* spp. show 2n = 38 (reviewed by Mariano et al. 2019; Barros et al. 2021). However, chromosomal fusion has been suggested as the origin of the derived karyotype from *Acromyrmexameliae* De Souza et al. 2007 (2n = 36) (Barros et al. 2021). In *Mycetophylax* Emery, 1913, both chromosomal fusions and fissions are important for the karyotypic evolution of species (Micolino et al. 2019a). In addition, other mechanisms that do not change the chromosome number were proposed for some species as differential heterochromatin growth in *Acromyrmex* spp. (Barros et al. 2016), duplications of euchromatic regions by unequal crossing-over or non-homologous translocations in *Mycetomoelleriusurichii* (Forel, 1893) (as *Trachymyrmexfuscus* Emery, 1934) (Barros et al. 2013a), paracentric inversion in *Acromyrmexechinatior* (Forel, 1899) (Barros et al. 2016; Teixeira et al. 2021) and pericentric inversion in *Mycetomoelleriusiheringi* (Emery, 1888) (Micolino et al. 2020).

There are different possible mechanisms involved in the karyotype evolution of Attina genera, highlighting the need to increase the number of studied species for more robust inferences (Barros et al. 2013b, 2018). The remaining genera of fungus-farming ants have little or no cytogenetically studied species; therefore, the processes that shaped their chromosomal evolution remain obscure. Therefore, using classical and molecular cytogenetic techniques, we determined the karyotypes of three fungus-farming ants – *Cyphomyrmextransversus* Emery, 1894, *Mycetomoelleriusrelictus* (Borgmeier, 1934), and *Sericomyrmexmaravalhas* Ješovnik et Schultz, 2017 – to investigate the presence of GC-rich heterochromatin in these species and understand the patterns of chromosomal evolution in their respective genera as well as in Attina in general.

## Material and methods

Colonies of *C.transversus*, *M.relictus*, and *S.maravalhas* were collected in Viçosa, in the Minas Gerais state, Brazil (-20.757041, -42.873516) (Table 1). Sampling permission was given by the Instituto Chico Mendes de Conservação da Biodiversidade (ICMBio) (SISBIO accession number 32459). Adult vouchers were identified by Dr. Jacques H. C. Delabie and deposited in the myrmecological collection of the Centro de Pesquisas do Cacau at the Comissão Executiva do Plano da Lavoura Cacaueira (CEPLAC), in Bahia, Brazil.

**Table 1.** Species of fungus-farming ants cytogenetically analyzed in the present study collected in Viçosa, Minas Gerais, Brazil. Species, total number of colonies and individuals; diploid chromosome numbers; diploid karyotype formulae, presence of GC-rich heterochromatin, and idiogram showing the location of 18S rDNA genes in the karyotype.

Species	Col. / Ind.	2n	Karyotype formulae	GC-rich Het	rDNA 18S location
*Cyphomyrmextransversus*	1 / 6	18	10m + 2sm + 6a	Yes	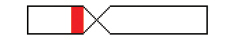
*Mycetomoelleriusrelictus*	2 / 7	20	20m	Yes	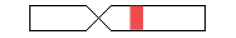
*Sericomyrmexmaravalhas*	2 / 14	48	28m + 20sm	Yes	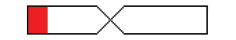

Mitotic metaphases were obtained from cerebral ganglia of larvae after meconium elimination accordingly to Imai et al. (1988). Chromosome number and morphology of metaphases were analyzed using conventional 4% Giemsa staining. Chromosomes were arranged in order of decreasing size, measured and classified according to the methodology proposed by Levan et al. (1964) that is based on the ratio of the chromosome arm lengths (r = long arm/short arm). The chromosomes were classified as m = metacentric (r = 1–1.7), sm = submetacentric (r = 1.7–3), st = subtelocentric (r = 3–7) and a = acrocentric (r > 7). Chromosomes were organized using Adobe Photoshop CS6 and measured using Image Pro Plus.

The heterochromatin distribution pattern was observed by C-banding technique according to Sumner (1972), with adaptations of Barros et al. (2013b). Metaphases were stained with the fluorochromes chromomycin A_3_ (CMA3) and 4’6-diamidino-2-phenylindole (DAPI), to the detection of GC and AT-rich regions, respectively based on the technique proposed by Schweizer (1980).

The ribosomal 18S gene clusters were detected by FISH, following the protocol of Pinkel et al. (1986) with the use of the 18S rDNA probes obtained via PCR amplification. The genomic DNA from the ant *Camponotusrufipes* (Fabricius, 1775) was used for amplification of 18S rDNA using the primers 18SF1 (5’-GTC ATA GCT TTG TCT CAA AGA-3’) and 18SR1.1 (5’-CGC AAA TGA AAC TTT TTT AAT CT-3’) (Pereira 2006). These primers amplify the initial portion of 18S rDNA (for details see Menezes et al. 2021). Gene amplification followed Pereira (2006). 18S rDNA probes were labeled by an indirect method using digoxigenin-11-dUTP (Roche Applied Science, Mannheim, Germany), and the FISH signals were detected with anti-digoxigenin-rhodamine (Roche Applied Science), following the manufacturer’s protocol.

Chromosomes from ten metaphases of each taxon were measured in order to determine the chromosomal morphology. For C-banding, fluorochrome staining, and FISH techniques, at least 30 metaphases of each taxon were analyzed. The metaphases were photographed using an epifluorescent microscope Olympus BX60 attached to an image system QColor Olympus with the filters WB (450–480 nm), WU (330–385 nm), and WG (510–550 nm) for the fluorochromes CMA_3_, DAPI, and rhodamine, respectively.

## Results

**Figure 1.**
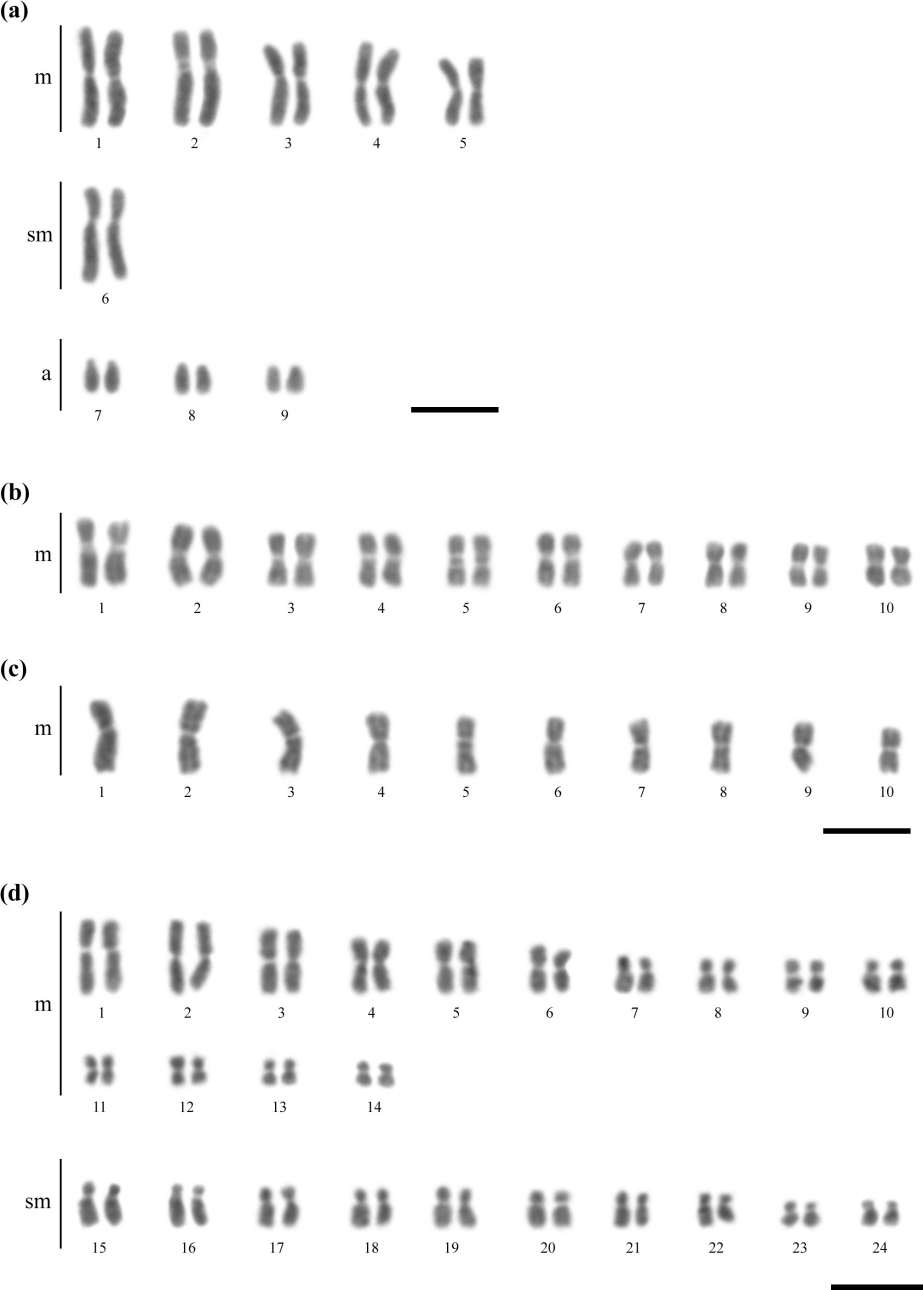
Karyotypes of fungus-farming ants **a***Cyphomyrmextransversus* (2n = 18, 10m + 2sm + 6a) **b, c***Mycetomoelleriusrelictus* (2n = 20, 20m and n = 10, 10m), and **d***Sericomyrmexmaravalhas* (2n = 48, 28m + 20sm). Scale bars: 5 µm.

The chromosome numbers and karyotypic formulae observed in the three fungus-farming ant species were as follows: 2n = 18 (10m + 2sm + 6a) in *C.transversus* (Fig. 1a), 2n = 20 (20m) and n = 10 (10m) in *M.relictus* (Fig. 1b, c), and 2n = 48 (28m + 20sm) in *S.maravalhas* (Fig. 1d).

**Figure 2.**
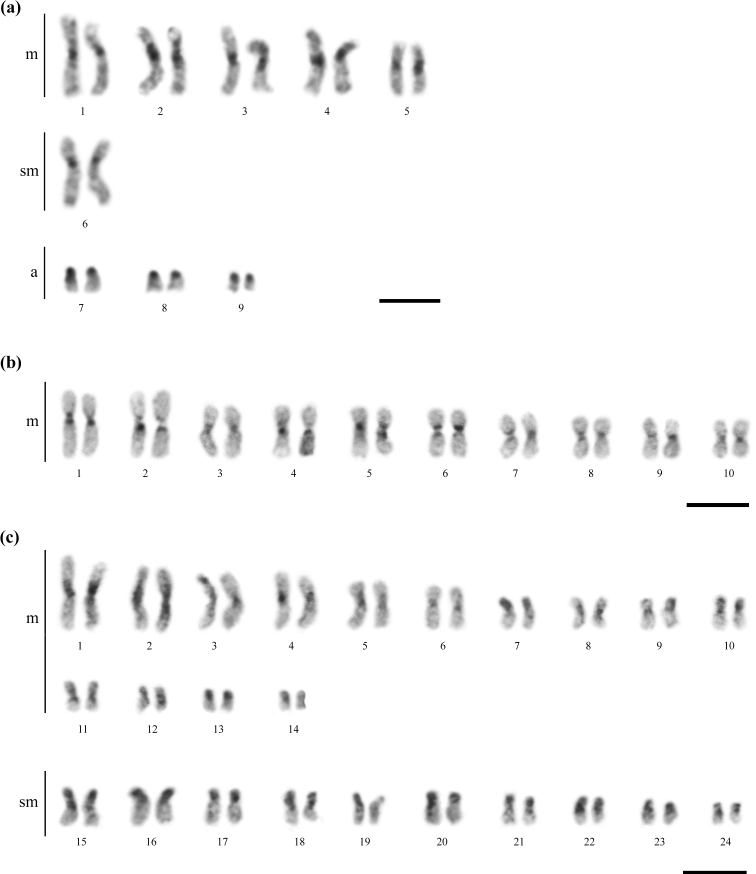
Heterochromatic patterns after C-banding technique in the karyotypes of the studied fungus-farming ants **a***Cyphomyrmextransversus* (2n = 18) **b***Mycetomoelleriusrelictus* (2n = 20), and **c***Sericomyrmexmaravalhas* (2n = 48). Dark blocks indicate heterochromatin in the centromeric/pericentromeric regions and short arms of the chromosomes. Scale bars: 5 µm.

**Figure 3.**
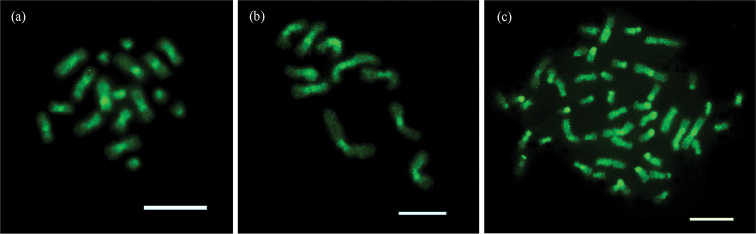
GC-rich chromatin patterns using Chromomycin A_3_ fluorochrome on metaphases of the studied fungus-farming ants **a***Cyphomyrmextransversus* (2n = 18) **b***Mycetomoelleriusrelictus* (n = 10), and **c***Sericomyrmexmaravalhas* (2n = 48). The GC-rich bands in the centromeric/pericentromeric regions and short arms of the chromosomes are colocalized with heterochromatic blocks. Scale bars: 5 µm.

Heterochromatin was observed in the centromeric/pericentromeric regions of all chromosomes besides short arms of acrocentric chromosomes in *C.transversus* (Fig. 2a). *Mycetomoelleriusrelictus* presented heterochromatic bands in the centromeric regions of all chromosomes (Fig. 2b). In *S.maravalhas*, heterochromatin was observed in the centromeric and pericentromeric regions of metacentric chromosomes, and short arms of the 7^th^, 10^th^, and 13^th^ metacentric and all submetacentric pairs (Fig. 2c). Most of the heterochromatic regions showed GC-rich patterns in all three species (Fig. 3).

**Figure 4.**
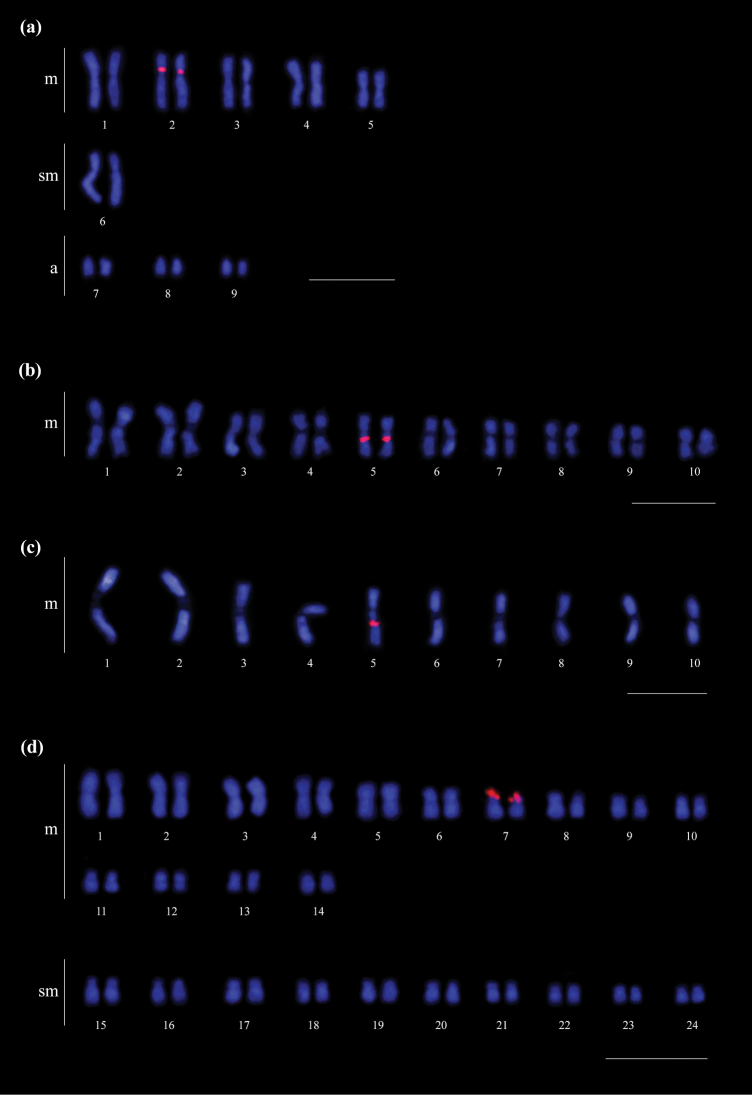
18S rDNA clusters (red blocks) location on the karyotypes of the studied fungus-farming ants **a***Cyphomyrmextransversus* (2n = 18) **b, c***Mycetomoelleriusrelictus* (2n = 20, n = 10), and **d***Sericomyrmexmaravalhas* (2n = 48). Scale bars: 5 µm.

The three species showed a single pair of chromosomes bearing rDNA clusters. The 18S ribosomal gene clusters were mapped in the pericentromeric region of the short arm of the 2^nd^ metacentric pair in *C.transversus* (Fig. 4a), in the interstitial region of the long arm of the 5^th^ metacentric pair in *M.relictus* (Fig. 4b, c), and in the terminal region of the short arm of the 7^th^ metacentric pair in *S.maravalhas* (Fig. 4d).

## Discussion

The association of cytogenetic and molecular data provided insights into the karyotype evolution of the three genera of fungus-farming ants in this study. In *Sericomyrmex*, the molecular phylogeny proposed by Ješovnik et al. (2017) showed that *S.maravalhas*, a new species recently described by Ješovnik and Schultz (2017), belongs to the *scrobifer* clade, which is basal to the other existing clade, the *amabilis*. Ješovnik and Schultz (2017) highlighted that the distribution data of *S.maravalhas* are clearly incomplete. This is the first report of this species in the Atlantic rainforest since its known occurrence, to this date, was restricted to Cerrado habitats.

*Sericomyrmexmaravalhas* (*scrobifer* clade) has a basal position to *Sericomyrmexamabilis* Wheeler, 1925 (*amabilis* clade) (Ješovnik et al. 2017). The former species has 2n = 48, with more submetacentric chromosomes (this study), whereas the latter species has 2n = 50 with only metacentric chromosomes (Murakami et al. 1998). It is possible to suggest an increase in chromosome number from 2n = 48 to 2n = 50. Additionally, the heterochromatic pattern on the short arms of the submetacentric/metacentric chromosomes of *S.maravalhas* is a strong indicator of centric fission events during the karyotype evolution in *Sericomyrmex*. The absence of subtelocentric/acrocentric chromosomes in the karyotype of *S.maravalhas*, which has also been observed in *S.amabilis* and *Sericomyrmex* sp. (Murakami et al. 1998; Barros et al. 2013b), can be associated with tandem growth of heterochromatin for telomeric stability after fission, which should have changed the chromosome’s morphology from acrocentric to submetacentric/metacentric. These events of heterochromatin growth may have contributed to differences in chromosomal morphology observed in *S.maravalhas* in relation to *Sericomyrmex* sp. and *S.amabilis*. A similar mechanism has also been suggested to explain interspecific chromosomal variations in leaf-cutting ants *Acromyrmex* (Barros et al. 2016) and trap-jaw ants *Odontomachus* (Aguiar et al. 2020).

The molecular phylogeny of *Mycetomoellerius*, proposed by Solomon et al. (2019), showed two main clades. One clade includes *M.urichii* with 2n = 18 chromosomes (Barros et al. 2013a), *Mycetomoelleriusholmgreni* (Wheeler, 1925), and *M.iheringi*, both of which have 2n = 20 chromosomes (Barros et al. 2018; Cardoso et al. 2018; Micolino et al. 2020; Table 2). *Mycetomoelleriusrelictus* belongs to the other clade and has 2n = 20 chromosomes (present study; Barros et al. 2013b). *Mycetomoellerius* sp. (as *Trachymyrmex* sp.) from the Atlantic rainforest has 2n = 22 (Barros et al. 2013b). Therefore, an ancestor of *Mycetomoellerius* with the chromosome number between 2n = 18–22 and with a predominance of metacentric chromosomes seems likely.

**Table 2.** Summary of available cytogenetic data in the literature and this study for the genera of fungus-farming ants *Cyphomyrmex*, *Sericomyrmex*, and *Mycetomoellerius*. Species, localities, chromosome numbers: diploid (2n)/haploid (n), diploid karyotype formulae, and references. The terminology used for karyotype formulae is in accordance to the published data.

Species	Localities	2n/(n)	Karyotype formulae	References
***Cyphomyrmex***				
*C.costatus*	Panama	20	20M	Murakami et al. (1998)
*C.cornutus*	French Guiana	22	10M + 12SM	Mariano et al. (2011)
*C.rimosus*	Panama	32	28M + 4A	Murakami et al. (1998)
*C.transversus*	French Guiana	24/(12)	14m + 6sm + 4a	Aguiar et al. (2020)
*C.transversus*	SP - Brazil	42	42A	Mariano et al. (2019)
*C.transversus*	MG - Brazil	18	10m + 2sm + 6a	**Present study**
*Cyphomyrmex* sp. §	MG - Brazil	32	14M + 18A	Mariano et al. (2019)
***Sericomyrmex***				
*S.amabilis*	Panama	50	50M	Murakami et al. (1998)
*S.maravalhas*	MG - Brazil	48	28m + 20sm	**Present study**
*Sericomyrmex* sp.	MG - Brazil	50/(25)	44m + 6sm	Barros et al. (2013b)
***Mycetomoellerius***				
M.urichii*	MG - Brazil	18	16m + 2sm	Barros et al. (2013a)
*M.holmgreni*	MG - Brazil	20	20m	Barros et al. (2018) / Cardoso et al. (2018)
*M.iheringi*	SC - Brazil	20	18M + 2SM	Micolino et al. (2020)
*M.relictus*	MG - Brazil	20/(10)	20m	Barros et al. (2013b) / **Present study**
*Mycetomoellerius* sp.†	MG - Brazil	22	18m + 4sm	Barros et al. (2013b)

* As *Trachymyrmexfuscus* in Barros et al. (2013a); † According to new revision by Solomon et al. (2019); § *Cyphomyrmex* sp. group *rimosus*. MG: Minas Gerais State; SP: São Paulo State; SC: Santa Catarina State.

The cytogenetic data obtained in this study for *C.transversus* (2n = 18) showed the lowest chromosome number for this genus. This karyotype is different from the other two previously studied karyotypes in French Guiana (2n = 24) and Brazil (2n = 42) (Mariano et al. 2019; Aguiar et al. 2020; Table 2). The chromosomal morphology also differs among the three karyotypes of *C.transversus*, with a notable increase in the number of acrocentric pairs in the karyotype from São Paulo-Brazil, which has a higher chromosome number. These data suggest that *C.transversus* may be a species complex and, therefore, cytogenetic data highlight the need for taxonomic revision of this species. Based on cytogenetic studies available for *Cyphomyrmex*, Mariano et al. (2019) suggested that centric fissions play a major role in the karyotype evolution within this genus due to an increase in acrocentric chromosome pairs in species with high chromosome numbers. Further molecular phylogenetic studies associated with cytogenetic data will help in the discussion of the karyotype evolution of this genus and the taxonomy of *C.transversus*.

Regarding heterochromatin constitution, the three species of the present study showed GC-rich heterochromatin, as evidenced by the colocalization of the heterochromatic and CMA_3_^+^ bands. These data were first reported in *Sericomyrmex* and *Cyphomyrmex*. Other fungus-farming ants showed the same heterochromatic composition such as *M.goeldii* (Paleoattina) (Barros et al. 2010), *M.urichii* (Barros et al. 2013a), and *M.holmgreni* (Barros et. al. 2018), included in Neoattina. This pattern is not common in ants, with few examples in the *Dolichoderus* genus, which belongs to another subfamily (Santos et al. 2016). Barros et al. (2018) suggested that GC-rich heterochromatin observed in different species of Attina, with representatives in Paleoattina and Neoattina, may have a common origin within the subtribe. The heterochromatic pattern rich in GC observed in this study supports this hypothesis, increasing the number of genera with this characteristic. Further investigation of the chromatin composition of these species should corroborate this hypothesis.

The physical mapping of rDNA genes showed a single chromosome pair bearing these genes for the three species in this study. This pattern is similar to that observed for other fungus-farming ants, which is suggested to be a plesiomorphic characteristic in Formicidae (reviewed by Teixeira et al. 2021), and aculeate Hymenoptera as well (Menezes et al. 2021). Regarding the location of these rDNA genes on the chromosomes in Attina, most species presented these genes in the intrachromosomal region (pericentromeric or interstitial). This characteristic is observed in ancient species such as *M.goeldii*, *Myrmicocrypta* sp., *Mycetophylax* spp., and *C.tranversus*, in the transition species *M.holmgreni* and *M.relictus*, and leaf-cutting ants, most derived from the group, *Am.striatus* and *Atta* spp. (reviewed by Teixeira et al. 2021; this study). These data suggest that the intrachromosomal position of rDNA genes seems to be a plesiomorphic character in fungus-farming ants.

**Figure 5.**
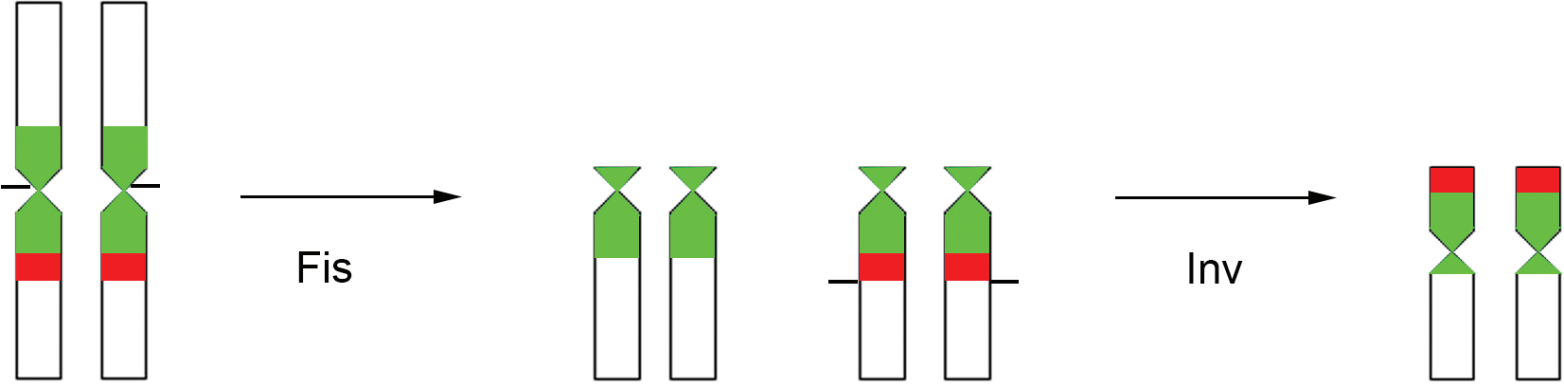
Diagram of origin of terminal rDNA clusters in metacentric chromosome from *Sericomyrmexmaravalhas*, considering its ancestor with intrachromosomal rDNA clusters. Black bars: chromosomal breaks; Fis: centric fission; Inv: pericentric inversion; Green blocks: GC-rich regions; Red blocks: 18S ribosomal clusters.

However, in *S.maravalhas*, the rDNA clusters were mapped in the terminal region of the heterochromatic short arm of the 7^th^ metacentric pair (see Figs 2c, 4c). Considering an ancestor with a low chromosome number and intrachromosomal rDNA clusters, after centric fission events, the occurrence of pericentric inversion would change the pericentromeric rDNA genes to the terminal positions, as observed in *S.maravalhas* (Fig. 5). In some other fungus-farming ants, rDNA genes are also located in the terminal region, such as *Acromyrmex* spp. (Barros et al. 2016; Teixeira et al. 2017), *Mycetophylaxconformis* (Mayr, 1884), and *M.morschi* (Emery, 1888) (2n = 30) (Micolino et al. 2019a), which are species with derived karyotypes within their respective phylogenetic branches. In the case of *M.conformis*, the terminal rDNA cluster located on the metacentric chromosome (Micolino et al. 2019a) seems to represent a derived pattern, explained by a single paracentric inversion, considering its ancestor with intrachromosomal rDNA clusters. The rDNA terminal location in *Acromyrmex* seems to be a derived condition among leaf-cutting ants (Barros et al. 2021).

In addition, a difference in the location of rDNA clusters was observed between *M.relictus* in this study and *M.holmgreni* (Barros et al. 2018; Micolino et al. 2019b). The former showed 18S rDNA clusters located in the interstitial region of the 5^th^ metacentric pair while the latter presented these genes in the pericentromeric region of the 4^th^ metacentric pair (Barros et al. 2018; Micolino et al. 2019b). This difference may reflect the phylogenetic position of these species, as they are included in distinct branches of *Mycetomoellerius*, in which *M.holmgreni* has a basal position to *M.relictus* (Solomon et al. 2019). However, the size variation between the 4^th^ and 5^th^ metacentric pairs was very subtle in *M.relictus* (see Fig. 4b, c). This suggests homeology of the chromosome pair carrying rDNA clusters between *M.relictus* and *M.holmgreni*. Therefore, the difference in the location of ribosomal genes between *M.relictus* and *M.homlgreni* may be the result of paracentric inversion. In addition, the occurrence of a paracentric inversion involving rDNA genes has already been observed in leaf-cutting ant *A.echinatior* (Teixeira et al. 2021). Thus, inversions seem to be important rearrangements that generate changes in the position of rDNA genes in the karyotype of fungus-farming ants.

## Conclusions

In this study, the distribution of 18S ribosomal genes and GC-rich heterochromatin in *Sericomyrmex* and *Cyphomyrmex*, which were reported for the first time, suggest the origin of this heterochromatin in the common ancestor of Attina. The karyotype observed in *C.tranversus* shows the lowest chromosomal number for the genus, and chromosomal variability among populations of the species highlights the need for taxonomic revision of this species using an integrative approach. Although *Sericomyrmex* spp. are morphologically complex (Ješovnik and Schultz 2017), karyotype differences were observed in this study, highlighting cytogenetics as an important tool for integrative taxonomy. Cytogenetic data obtained for *S.maravalhas* suggested centric fission events during chromosomal evolution in *Sericomyrmex*. Inversions seem to be involved in the origin of location of 18S ribosomal genes in *M.relictus* and *S.maravalhas*. Therefore, this study provides new insights into chromosomal evolution in *Sericomyrmex*, *Cyphomyrmex*, and *Mycetomoellerius*. Our data suggest that chromosomal rearrangements have contributed to the species diversification in Attina. We also believe that the increase in the number of species studied using classical and molecular cytogenetic techniques will continue to contribute to discussions about the evolution of fungus-farming ants.
